# Supplementary material for: Experimental quantum simulation of superradiant phase transition beyond no-go theorem via antisqueezing
Source: Nat Commun. 2021 Nov 1;12:6281. doi: 10.1038/s41467-021-26573-5 (PMC8560888; doi:10.1038/s41467-021-26573-5)
Supplement: Supplementary file 1 — Supplementary Information [file 41467_2021_26573_MOESM1_ESM.pdf]

Supplementary Information-Experimental quantum simulation of superradiant phase transition beyond no-go theorem via antisqueezing

Xi Chen,[1,2,3,a)] Ze Wu,[1,2,3,a)] Min Jiang,[1,2,3] Xin-You Lü,[4,b)] Xinhua Peng,[1,2,3,c)] and Jiangfeng Du[1,2,3]

[1)] *Hefei National Laboratory for Physical Sciences at the Microscale and Department of Modern Physics, University of Science and Technology of China, Hefei 230026, China*

[2)] *CAS Key Laboratory of Microscale Magnetic Resonance, University of Science and Technology of China, Hefei 230026, China*

[3)] *Synergetic Innovation Center of Quantum Information and Quantum Physics, University of Science and Technology of China, Hefei 230026, China*

[4)] *School of physics, Huazhong University of Science and Technology, Wuhan 430074, China*

This supplementary information contains six parts: 1. The detailed derivation of superradiant phase transition (SPT) beyond no-go theorem induced by the antisqueezing effects; 2. We analytically present the singularity of quantum fluctuation at the critical point, and the generation of quantum superposition associating with the SPT; 3. The additional experiments and analyses for supporting the nontrivial SPT obtained in our experiment; 4. The implementation of squeezing operator with the platform of NMR is discussed; 5. We present the detailed procedures of the initial state preparation; 6. We show the detailed measurement processes for zero point fluctuation (ZPF) and the order parameter; 7. We analyze the experimental errors and the mechanism of ZPF enhancement.

Supplementary Note 1. Analytical derivation of antisqueezing induced SPT

In our work, the simulated cavity QED system including A^2 and antisqueezing terms is described by Hamiltonian $\hat{\mathbb{H}} = \hat{H}_R + \hat{H}_A + \hat{H}_{As}$, given by

$$\begin{aligned} \hat{H}_R &= \frac{\Omega}{2}\hat{\sigma}_z + \omega\hat{a}^\dagger\hat{a} + \lambda\left(\hat{a}^\dagger + \hat{a}\right)\hat{\sigma}_x, \\ \hat{H}_A &= \alpha\frac{\lambda^2}{\Omega}\left(\hat{a} + \hat{a}^\dagger\right)^2, \\ \hat{H}_{As} &= -\xi\left(\hat{a} + \hat{a}^\dagger\right)^2, \end{aligned} \tag{1}$$

where $\hat{H}_R$ is the standard Rabi Hamiltonian, and $\hat{H}_A$ refers to the so-called A^2-term that prevents the occurrence of equilibrium SPT in normal cavity QED systems. Interestingly, $\hat{H}_{As}$, corresponding to an antisqueezing operation, is the key term to recover SPT in the case of including A^2 term. Similar as the case of standard Rabi model[1], Hamiltonian of system $\hat{\mathbb{H}}$ can be analytically diagonalized in the classical oscillator limit $\Omega/\omega \to \infty$, and the corresponding solutions offer the guidance of implementing our experiment shown in the main text.

Applying a squeezing transformation $\hat{S}(\tilde{r}) = \exp\left[\tilde{r}(\hat{a}^2 - \hat{a}^{\dagger 2})/2\right]$ with squeezing parameter

$$\tilde{r} = \frac{1}{4}\ln\left(1 + \alpha\tilde{\lambda}^2 - 4\frac{\xi}{\omega}\right), \quad \tilde{\lambda} = \frac{2\lambda}{\sqrt{\Omega\omega}}, \tag{2}$$

into the system Hamiltonian, we obtain

$$\hat{\mathbb{H}}_s = \hat{S}^\dagger(\tilde{r})\,\hat{\mathbb{H}}\hat{S}(\tilde{r}) = \frac{\Omega}{2}\hat{\sigma}_z + \omega_s\hat{a}^\dagger\hat{a} + \lambda_s\left(\hat{a}^\dagger + \hat{a}\right)\hat{\sigma}_x + C_s, \tag{3}$$

where

$$\omega_s = \omega e^{2\tilde{r}}, \quad \lambda_s = \lambda e^{-\tilde{r}}, \quad C_s = \left(e^{2\tilde{r}} - 1\right)\frac{\omega}{2}. \tag{4}$$

Note that, the ground state of Hamiltonian $\hat{\mathbb{H}}$ is equivalent to $\hat{S}(\tilde{r})\,|G\rangle_s$, where $|G\rangle_s$ is the ground state of Hamiltonian $\hat{\mathbb{H}}_s$. Now, the effects from A^2 term and antisqueezing operation have been introduced into the system parameters ω_s and λ_s, and then

---

[a)] These authors contributed equally to this work
[b)] Electronic mail: xinyoulu@hust.edu.cn
[c)] Electronic mail: xhpeng@ustc.edu.cn

the property of SPT can be obtained by experimentally searching the ground state of $\mathbb{H}_s$, which can be diagonalized in the limit $\Omega/\omega_s \to \infty$, corresponding to $\Omega/\omega \to \infty$ in terms of the original system parameter.

When $\tilde{\lambda} < \exp(2\tilde{r}) = \sqrt{1+\alpha\tilde{\lambda}^2 - 4\xi/\omega}$, i.e., $\tilde{\lambda} < \sqrt{\frac{4\xi/\omega-1}{\alpha-1}}$, the system is in the normal phase (NP). Applying a unitary transformation with

$$\hat{U} = \exp\left[\frac{\lambda_s}{\Omega}\left(\hat{a}+\hat{a}^\dagger\right)(\hat{\sigma}_+ - \hat{\sigma}_-)\right], \tag{5}$$

we obtain

$$\hat{U}^\dagger \hat{\mathbb{H}}_s \hat{U} = \omega_s \hat{a}^\dagger \hat{a} + \frac{\tilde{\lambda}_s^2 \omega_s}{4}(\hat{a}^\dagger + \hat{a})^2 \hat{\sigma}_z + \frac{\Omega}{2}\hat{\sigma}_z + C_s, \tag{6}$$

where $\tilde{\lambda}_s = 2\lambda_s/\sqrt{\Omega\omega_s}$. Then, projecting the system into the spin-down subspace, the Hamiltonian becomes

$$\hat{\mathbb{H}}_s^{\text{np}} = \omega_s \hat{a}^\dagger \hat{a} - \frac{\tilde{\lambda}_s^2 \omega_s}{4}(\hat{a}^\dagger + \hat{a})^2 - \frac{\Omega}{2} + C_s, \tag{7}$$

which can be fully diagonalized through another squeezing transformation

$$\hat{S}^\dagger\left(l_{\text{np}}\right)\hat{\mathbb{H}}_s^{\text{np}}\hat{S}\left(l_{\text{np}}\right) = \omega_e \hat{a}^\dagger \hat{a} + E_g. \tag{8}$$

Here the squeezing operator $\hat{S}\left(l_{\text{np}}\right) = \exp\left[l_{\text{np}}\left(\hat{a}^2 - \hat{a}^{\dagger 2}\right)/2\right]$ with squeezing parameter $l_{\text{np}} = \ln\left(1-\tilde{\lambda}_s^2\right)/4$. The excitation energy ω_e and the ground state energy E_g are given by

$$\begin{aligned} \omega_e &= \omega_s\sqrt{1-\tilde{\lambda}_s^2} \\ E_g &= \frac{\omega_s}{2}\left(\sqrt{1-\tilde{\lambda}_s^2}-1\right) - \frac{\Omega}{2} + C_s. \end{aligned} \tag{9}$$

The corresponding eigenstates of the system $\hat{\mathbb{H}}$ are

$$|G\rangle_{\text{np}} = \hat{S}(\tilde{r}_{\text{np}})\,|m\rangle\,|\downarrow\rangle, \tag{10}$$

and $\tilde{r}_{\text{np}} = \tilde{r} + l_{\text{np}}$. It should be noted that the excitation energy ω_e is real only for $\tilde{\lambda} < \exp(2\tilde{r})$ and vanishes when $\tilde{\lambda} = \exp(2\tilde{r})$, which is the typical sign of the occurrence of SPT.

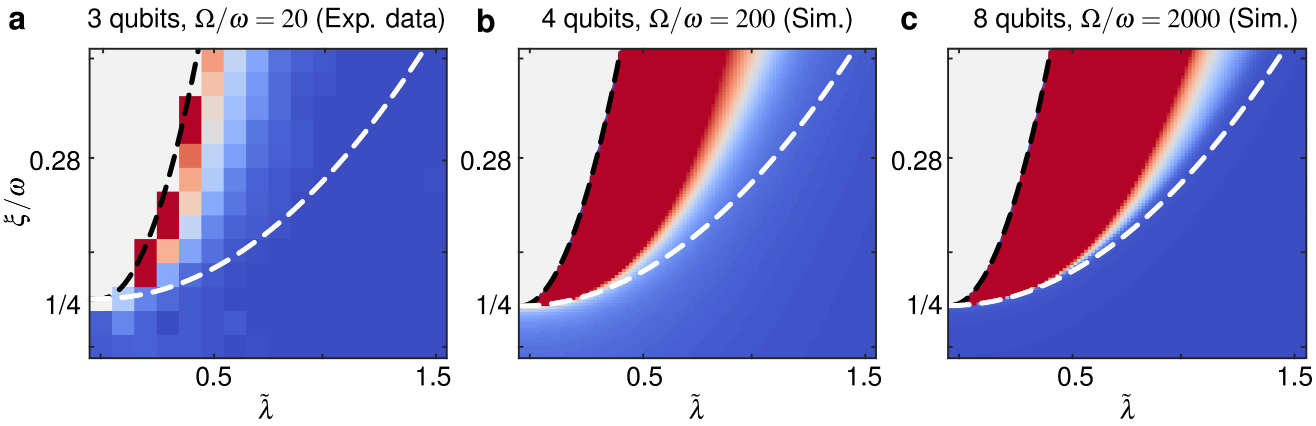

Supplementary Figure 1. **Asymptotic behavior of the order parameter**. **a,** The dependence of experimental Φ on $\tilde{\lambda}$ and ξ/ω for $\Omega/\omega = 20$, which is exactly Fig. 4**b** of main text. **b,** The numerical simulation on 4 qubits and $\Omega/\omega = 200$. **c,** The numerical simulation on 8 qubits and $\Omega/\omega = 2000$. The results demonstrate that the border of superradiant phase is gradually approaching to the exact critical line (i.e., the white dashed line) when Ω/ω and the simulating space becomes larger.

When $\tilde{\lambda} > \exp(2\tilde{r}) = \sqrt{1+\alpha\tilde{\lambda}^2 - 4\xi/\omega}$, i.e., $\tilde{\lambda} > \sqrt{\frac{4\xi/\omega-1}{\alpha-1}}$, the system enters into the superradiant phase (SP), and the Hamiltonian $\hat{\mathbb{H}}_s^{\text{np}}$ becomes invalid since the boson mode $\hat{a}$ are macroscopically excited. Then we should firstly apply the displacement transformation into Hamiltonian $\hat{\mathbb{H}}_s$ and obtain

$$\hat{D}^\dagger(\beta)\hat{\mathbb{H}}_s^{\text{np}}\hat{D}(\beta) = \frac{\Omega'}{2}\hat{\sigma}_z' + \omega_s\hat{a}^\dagger\hat{a} + \lambda_s\left(\hat{a}^\dagger + \hat{a}\right)\hat{\sigma}_x' + \omega_s\beta^2 + C_s, \tag{11}$$

where $\Omega' = \tilde{\lambda}_s^2\Omega$, $\hat{D}(\beta) = \exp\left(\beta\hat{a}^\dagger - \beta^*\hat{a}\right)$ with $\beta = \pm\sqrt{\Omega\left(\tilde{\lambda}_s^2 - \tilde{\lambda}_s^{-2}\right)/4\omega_s}$, and the rotated Pauli operators $\hat{\sigma}_z'$, $\hat{\sigma}_x'$ are given by $|\downarrow\rangle' = \cos\theta|\downarrow\rangle - \sin\theta|\uparrow\rangle$ and $|\uparrow\rangle' = \sin\theta|\downarrow\rangle + \cos\theta|\uparrow\rangle$ with $\tan(2\theta) = -4\lambda_s\beta/\Omega$. Hamiltonian (11) is similar as Hamiltonian $\mathbb{H}_s$, and then it can be diagonalized by employing the similar procedure used in the normal phase. The corresponding excitation energy ω_e' and ground state energy E_g' becomes

$$\begin{aligned} \omega_e' &= \omega_s\sqrt{1-\tilde{\lambda}_s^{-4}} \\ E_g' &= \frac{\omega_s}{2}\left(\sqrt{1-\tilde{\lambda}_s^{-4}}-1\right) - \frac{\Omega}{4}(\tilde{\lambda}_s^2+\tilde{\lambda}_s^{-2}) + C_s. \end{aligned} \tag{12}$$

The ground state of system becomes twofold degenerate, i.e.,

$$|G\rangle_{\rm sp}^{\pm} = \hat{S}(\tilde{r})\hat{D}(\pm|\beta|)\hat{S}(l_{\rm sp})\,|0\rangle_a\,|\downarrow\rangle_\pm \approx \hat{S}(\tilde{r})\hat{D}(\pm|\beta|)\,|0\rangle_a\,|\downarrow\rangle_\pm\,, \tag{13}$$

where $l_{\rm sp} = (1/4)\ln\left(1-\tilde{\lambda}_s^{-4}\right)$ approaches zero in the parameter regime chosen in our experiment, and

$$|\downarrow\rangle_\pm = \sqrt{\frac{1}{2}\left(1+\tilde{\lambda}_s^{-2}\right)}\,|\downarrow\rangle \pm \sqrt{\frac{1}{2}\left(1-\tilde{\lambda}_s^{-2}\right)}\,|\uparrow\rangle\,. \tag{14}$$

Then the ground state of system in the superradiant phase can be written as

$$|G\rangle_{\rm sp} \approx \frac{1}{\sqrt{2}}\hat{S}(\tilde{r})\left[\hat{D}(|\beta|)\,|0\rangle_a\,|\downarrow\rangle_+ + \hat{D}(-|\beta|)\,|0\rangle_a\,|\downarrow\rangle_-\right]. \tag{15}$$

The SPT also can be characterized by the sudden change of the rescaled ground-state occupation of field $\hat{a}$, i.e., $\Phi = (\omega/\Omega)\langle\hat{a}^\dagger\hat{a}\rangle_g$. Based on the above analytical results in the classical oscillator limit $\Omega/\omega \to \infty$, $\Phi = 0$ when the system is in the normal phase $\left(\tilde{\lambda} < \sqrt{1+\alpha\tilde{\lambda}^2-4\xi/\omega}, \tilde{\lambda} > \sqrt{\frac{4\xi/\omega-1}{\alpha-1}}\right)$ and $\Phi = (e^{-4\tilde{r}}/4)(\tilde{\lambda}_s^2 - \tilde{\lambda}_s^{-2})$ becomes non-zero when it enters into the superradiant phase $\left(\tilde{\lambda} > \sqrt{1+\alpha\tilde{\lambda}^2-4\xi/\omega}, \tilde{\lambda} < \sqrt{\frac{4\xi/\omega-1}{\alpha-1}}\right)$. Associating with the SPT, there is a spontaneously $\mathbb{Z}_2$ symmetry breaking, i.e., the $\mathbb{Z}_2$ symmetry of the ground state is broken in the superradiant phase. It can be demonstrated by the ground-state coherence of field $\langle\hat{a}\rangle_g$, and $\langle\hat{a}\rangle_g$ changes from 0 to $\pm\exp(-\tilde{r})|\beta|$ when the system enters into the superradiant phase from normal phase. Besides the normal and superradiant phases, the system will become unstable when ω_s and Φ becomes the imaginary numbers in the regime of $1+\alpha\tilde{\lambda}^2-4\xi/\omega < 0$, corresponding to $\tilde{\lambda} < \sqrt{(1/\alpha)(4\xi/\omega-1)}$. In a short summary, the order parameter Φ will sudden changes at the critical lines $\tilde{\lambda} = \sqrt{1+\alpha\tilde{\lambda}^2-4\xi/\omega}$ and $\tilde{\lambda} = \sqrt{(1/\alpha)(4\xi/\omega-1)}$, which divides the NP, SP and unstable phase (UP). In the finite parameter regime, the dependence of Φ on the system parameters approaches the case of exact phase transition along with increasing Ω/ω, and this asymptotic behavior is clearly shown in **Supplementary Figure 1**.

Supplementary Note 2. ZPF singularity and quantum superposition associating with the SPT

The above results show that the antisqueezing effect could induce the occurrence of SPT even in the case of including A^2 term. This essentially comes from the recovering of singularity of quantum fluctuation at the critical point induced by the antisqueezing effects. Based on the analytical solutions in Section I, this singularity property could be demonstrated by calculating the ZPF of system. Firstly, the variance of quadrature $\hat{x} = \left(\hat{a}+\hat{a}^\dagger\right)/2$ in the ground state of Rabi model with Hamiltonian $\hat{H}_R$ is

$$\mathrm{ZPF}_1 \equiv \sqrt{\left\langle\hat{x}^2\right\rangle - \langle\hat{x}\rangle^2} = \begin{cases} (1/2)\left(1-\tilde{\lambda}^2\right)^{-1/4} & (\tilde{\lambda} < 1)\,, \\ (1/2)\left(1-\tilde{\lambda}^{-4}\right)^{-1/4} & (\tilde{\lambda} > 1)\,, \end{cases} \tag{16}$$

which corresponds to the black solid line of **Supplementary Figure 2**a. Secondly, including the A^2 term, the Hamiltonian of system becomes $\hat{H}_R + \hat{H}_A$, and the ZPF is

$$\mathrm{ZPF}_2 = \frac{1}{2}\left(1+\alpha\tilde{\lambda}^2-\tilde{\lambda}^2\right)^{-\frac{1}{4}}, \tag{17}$$

which is the blue dashed line of **Supplementary Figure 2**a, and clearly demonstrates that the singularity of ZPF disappears, corresponding to the no-go theorem. Lastly, with the antisqueezing term H_{As} included, the ZPF of system with Hamiltonian $\hat{H}_R + \hat{H}_A + \hat{H}_{As}$ becomes

$$\mathrm{ZPF}_3 = \begin{cases} (1/2)\left(1-\tilde{\lambda}_s^2\right)^{-1/4}\left(1+\alpha\tilde{\lambda}^2-4\xi/\omega\right)^{-1/4} & (\tilde{\lambda} < \tilde{\lambda}_c)\,, \\ (1/2)\left(1-\tilde{\lambda}_s^{-4}\right)^{-1/4}\left(1+\alpha\tilde{\lambda}^2-4\xi/\omega\right)^{-1/4} & (\tilde{\lambda} > \tilde{\lambda}_c)\,, \end{cases} \tag{18}$$

which corresponds to the red dot-dash line in **Supplementary Figure 2**a, and clearly shows that the singularity of ZPF is recovered. Here $\tilde{\lambda}_c = \sqrt{(4\xi/\omega - 1)/(\alpha - 1)}$, obtained by $\tilde{\lambda} = \sqrt{1 + \alpha\tilde{\lambda}^2 - 4\xi/\omega}$. This results also certify the main methodology of this work that the antisqueezing effect can break through the no-go theorem of SPT.

Associating with the SPT, quantum entanglement and quantum superposition occur in the superradiant phase. In the limit $\Omega/\omega \to \infty$, the ground state of Hamiltonian $\mathbb{H}$ is theoretically predicted as a spin-field entangled state in the superradiant phase as Eq. (15) shows. From this entangled ground state, we also can obtain the Schrödinger cat states of field $\hat{a}$ by measuring the spin in the $(|\downarrow\rangle_+ \pm |\uparrow\rangle_-)/\sqrt{2}$ basis. Depending on the outcome of the measurement, the state of simulated boson field is approximately projected into one of the following squeezed cat states

$$|\Psi\rangle_{\mathrm{cat}} \approx \frac{1}{\sqrt{2}}\hat{S}(\tilde{r})\left[\hat{D}(|\beta|)|0\rangle_a \pm \hat{D}(-|\beta|)|0\rangle_a\right]. \tag{19}$$

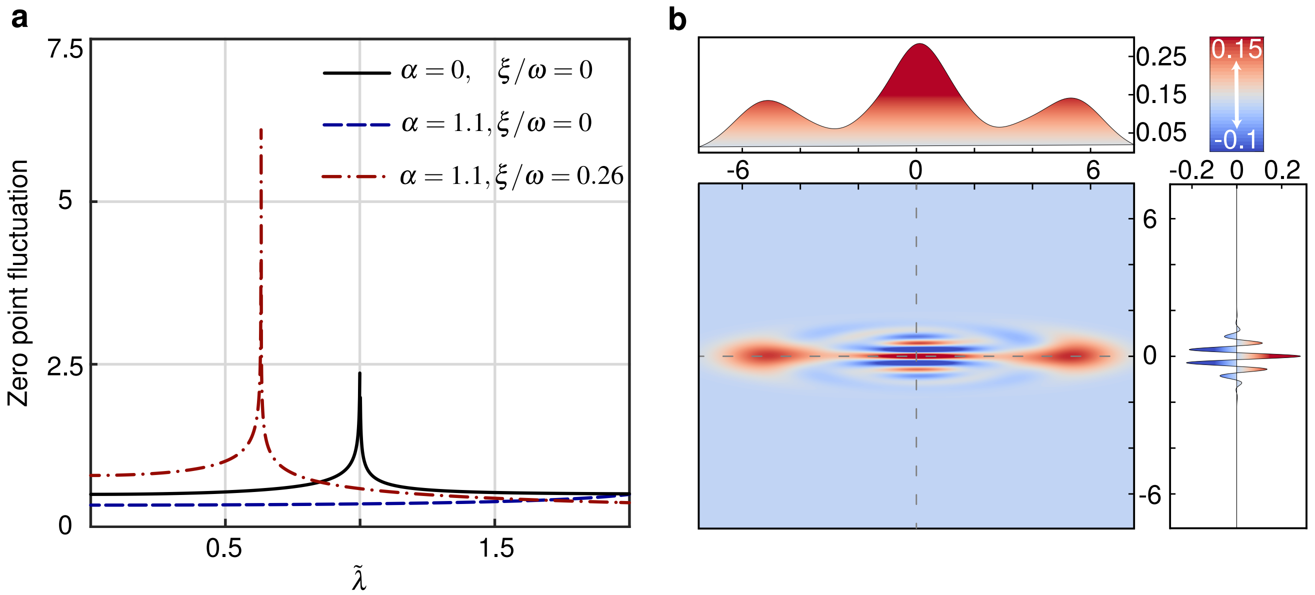

Supplementary Figure 2. **Antisqueezing recovering the singularity of ZPF and experimental Schrödinger cat states in SP. a,** The black line demonstrates the singularity of Rabi model while the blue line means the disappearance of the singularity because of no-go term, and the antisqueezing effect recovers it as shown by the red line. **b,** The Wigner functions of experimental reconstructed ground state with system parameters $\Omega/\omega = 25$, $\tilde{\lambda} = 0.3$.

To show the Schrödinger cat states clearly, we plot the Wigner functions of the ground state prepared experimentally. By the quantum state tomography process, we could obtain a 16×16 density matrix for the four-spin sample. And the Wigner functions in Fig. 3c were obtained from the reduced density matrix of the bosonic field by tracing out the two-level system, i.e., a 8×8 density matrix as shown in Fig. 4c. By employing the QuTiP package in Python, we could calculate the Wigner functions from the matrix representation of states in the truncated Fock basis, which are experimentally reconstructed by state tomography. Here we have used the Wigner transform of any matrix element $|n\rangle\langle m|$, i.e.,

$$f_{mn} = \sqrt{\frac{m!}{n!}} e^{i(m-n)\arctan(p/x)} \frac{(-1)^m}{\pi\hbar}\left(\frac{x^2+p^2}{\hbar/2}\right)^{(n-m)/2} L_m^{n-m}\left(\frac{x^2+p^2}{\hbar/2}\right) e^{-(x^2+p^2)/\hbar}, \tag{20}$$

where L_m^n is the associated Laguerre polynomials. By this, the Wigner function of any quantum state like $\rho = \sum_{n,m} r_{n,m} |n\rangle\langle m|$ can be easily achieved. In **Supplementary Figure 2**b, we plot the Wigner function of the experimental reconstructed ground state, which is a clear squeezed Schrödinger cat state as the theoretical prediction. Also, the obtained Schrödinger cat state has the negative Wigner distribution and distinct interference fringes. Two superposed coherent states separate with a large distance in phase space. This means it offers good quantum resources for quantum precision measurement and quantum information processing. Note that the above method for plotting Wigner function is also used in the main text. In the real bosonic systems, Wigner functions can be directly measured by the photon numbers[2]. But for a multi-spin system like our case, tomographic reconstruction is also widely used[3].

Supplementary Note 3. Additional experiments and analyses of the antisqueezing-induced SPT

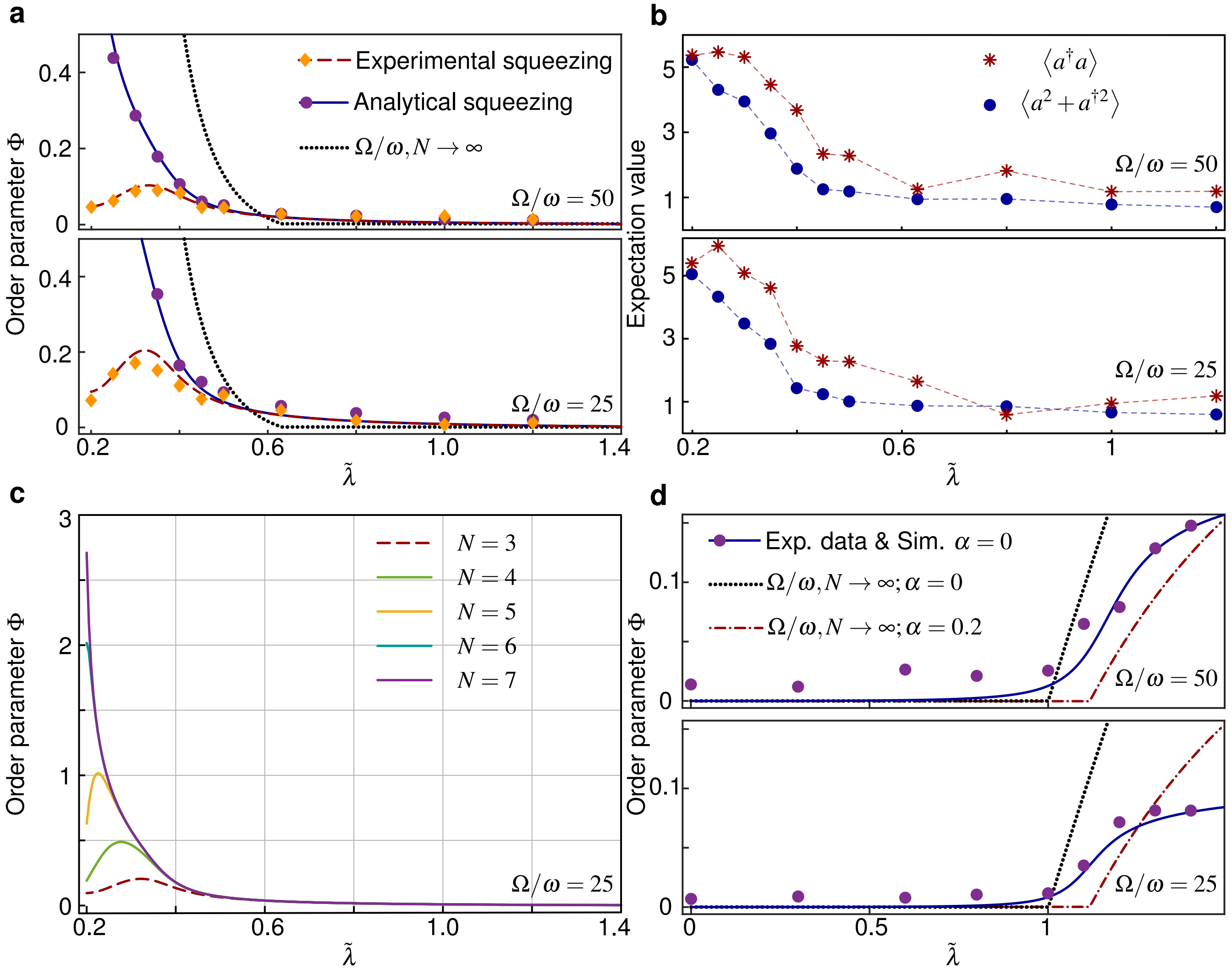

Supplementary Figure 3. **Additional experiments and numerical simulations on the SPT in different cases. a,** The dependences of order parameter Φ on $\tilde{\lambda}$ with and without the implementation of the squeezing operation, and the theoretical prediction in the classical oscillator limit. Here the diamonds and circles are the experimental data, and the upper (down) is corresponding to system parameters $\Omega/\omega = 50$ ($\Omega/\omega = 25$) and $\xi/\omega = 0.26$. **b,** The raw data of $\langle a^\dagger a \rangle_s$ and $\langle a^2 + a^{\dagger 2} \rangle_s$, which correspond to the data points in Subfigure a. **c,** Numerical simulation of the SPT behaviors with the truncated squeezing operator of different-size Hilbert space, and the numerical dimensions of squeezing operators are from 2^3 to 2^7. **d,** The dependences of order parameter Φ on $\tilde{\lambda}$ for the case of standard Rabi model with A^2 term at different intensities. The upper (down) is corresponding to the system parameter $\Omega/\omega = 50$ ($\Omega/\omega = 25$). The black (red) dotted lines are theoretical order parameters in the limit $\Omega/\omega \to \infty$ and $N \to \infty$ with $\alpha = 0$ ($\alpha = 0.2$). The circles are the experimental data for $\alpha = 0$.

Two sets of experiments were designed to simulate the SPT behaviors with limited qubits. Namely, we have realized the ground state simulation of $\hat{\mathbb{H}}$ with and without the implementation of the squeezing operation, and the experimental data are shown in Supplementary Figure 3a. Since the truncated squeezing operator in small Hilbert space with large squeezing parameter r will loss the validity (see Supplementary Figure 4), the order parameter in deep superradiant phase drops down. To illustrate the effect of the truncated squeezing operator more clearly, we numerically simulate the behaviors of the SPT with the truncated squeezing operator of different-size Hilbert space, showing the disappearance of the decreasing behavior in deep superradiant phase when the large enough Hilbert space is used (see Supplementary Figure 3c). So, in order to demonstrate the occurrence of the SPT in an effective way with the limited qubits, we adopt an alternative experimental scheme by means of the post-processing. The final experimental results, i.e., the order parameter of $\hat{\mathbb{H}}$ is reconstructed from two parts of the expectation values under the ground state of $\hat{\mathbb{H}}_s$, i.e., $\langle a^\dagger a \rangle_s$ and $\langle a^2 + a^{\dagger 2} \rangle_s$, as shown in Eq. (13) of the main text. Now we have plotted the raw data of

$\langle a^\dagger a \rangle_s$ and $\langle a^2 + a^{\dagger 2} \rangle_s$ in **Supplementary Figure 3**b. These data without post-processing is not just the order parameters of QRM without the A^2 term, and the SPT of $\hat{\mathbb{H}}$ can never be obtained with only the experimental results of QRM without A^2 term, i.e.,$\langle a^\dagger a \rangle_s$.

The antisqueezing induced SPT obtained in our work is quite different from both the cases of standard Rabi model and Rabi model with A^2 term at weak strength, i.e., $\alpha < 1$. First of all, our work is different from the recent experiment of simulating normal SPT in the standard Rabi model (i.e., $\alpha = 0$ and $\xi = 0$) with trapped-ion system[4]. Note that this normal SPT is limited by the no-go theorem, i.e., it will disappear when the A^2 term with $\alpha > 1$ is included[5]. Here we mainly demonstrate experimentally the recovering of SPT in the case of including A^2 term by introducing antisqueezing effect in the platform of NMR. In other words, the antisqueezing effect could induce the occurrence of SPT beyond no-go theorem. When $\alpha = 0$ and $\xi = 0$, our model is reduced to the case of standard Rabi model $\hat{H}_R$. To clearly show the difference between our work and the work of M. L. Cai et, al[4], we also experimentally demonstrated the SPT of $\hat{H}_R$ using our four qubit NMR simulator, as shown in **Supplementary Figure 3**d. In the experiment, we first adiabatically prepare the ground state of Hamiltonian $\hat{H}_R$ and then measure the order parameter $\Phi = (\omega/\Omega)\langle \hat{a}^\dagger \hat{a} \rangle$ according to Eq. (5) in the main text, and the results are similar to the SPT shown by M. L. Cai et, al. Comparing Figs. **Supplementary Figure 3**a and d, it is clearly shown that the nontrivial competition between the A^2 term and antisuqeezing term not only recovers the SPT, but also makes the SPT *reversed*, i.e., the phase transition to the superradiant phase happens as decreasing the spin-field coupling strength. Note that the *reversed* SPT has not been experimentally reported before. Moreover, associating with the reversed SPT, we experimentally observe the quantum entanglement and quantum superposition at the weak spin-field coupling strength, as shown in the main text.

Moreover, our work is also different from the case of simulating SPT in the Rabi model with A^2 term at weak strength, i.e., $\alpha < 1$. In our proposal, the antisqueezing term does not simply reduce the strength of A^2 term. This is because the antisqueezing term and A^2 term are different physically. The introduced antisqueezing term provides a modification on the potential of the bosonic field with fixed strength, i.e., being λ independent. However, the A^2 term is λ dependent, which means its strength is altered when one checks the occurrence of QPT by changing the spin-field coupling strength λ. Thus, the influence of them on the SPT can **not** be simply understood as the plus or minus of them. To make it more clear, we numerically simulate the SPT in the Rabi model with A^2 term and $\alpha = 0.2$ in **Supplementary Figure 3**d. It is similar to the normal SPT, and clearly different from the *reversed* SPT realized in our proposal. The direction of happening phase transition as changing spin-field coupling is opposite to the antisqueezing induced SPT obtained in our work.

Lastly, the physical mechanism of the antisqueezing recovering SPT demonstrated in our work is nontrivial. As shown in the above section, the antisqueezing effect recovers the SPT in the case of including A^2 term by increasing the ZPF of system and recovering its singularity. Associating with the appearance of the singularity of ZPF, quantum fluctuation drives the recovering of SPT at the critical point.

Supplementary Note 4. Validity of squeezing operator

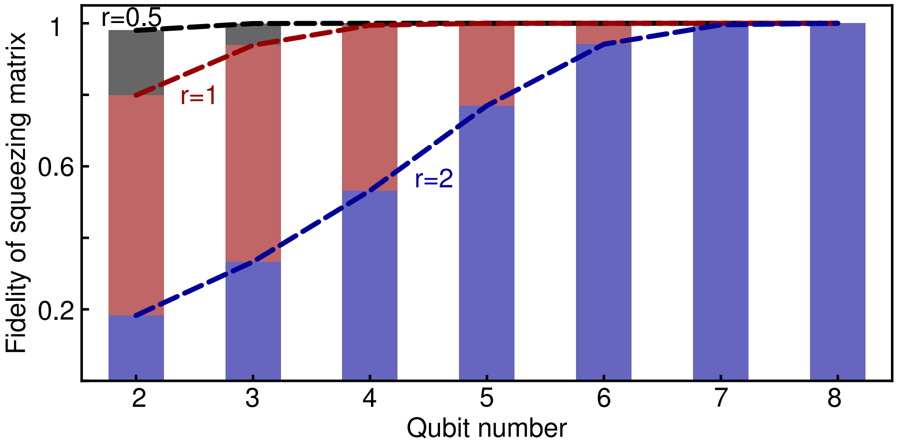

Supplementary Figure 4. **Validity of the truncated squeezing operator.** The fidelities of the squeezing operator vs the used qubit number for different squeezing parameters r. Here 10-qubit squeezing operator is chosen as the target matrix.

The antisqueezing effect plays a pivotal role in recovering SPT with the presence of A^2 term. Based on the defined mapping scheme in the Methods of the main text, the truncated squeezing matrices in the 2^N dimensional Hilbert space can be mapped

by using N spins, i.e., $\hat{S}(r) = \exp\left[r(\hat{a}^2 - \hat{a}^{\dagger 2})/2\right]$, where $\hat{a}$ and $\hat{a}^\dagger$ are given by Eq. (3) of main text. We have

$$\hat{a}^2 - \hat{a}^{\dagger 2} = \sum_{n=0}^{2^N-1} \sqrt{(n+1)(n+2)}\, |n\rangle\langle n+2| - h.c. \tag{21}$$

In the antisqueezing-enhanced ZPF experiment in the main text, the truncated squeezing matrices are implemented by NMR pulse sequences with time 15 ms, which are optimized by gradient ascent pulse engineering (GRAPE) method.

To identify quantitatively the valid regime of the mapped squeezing operator, we define the fidelity of the truncated squeezing operator as $F = \langle\psi|\hat{S}_T^\dagger(r)\hat{S}(r)|\psi\rangle$, where $\hat{S}_T(r)$ and $\hat{S}(r)$ are the target matrix and the mapped matrix with N spins, respectively. In principle, $|\psi\rangle$ is any pure state of boson field $\hat{a}$, and here we choose the vacuum state for simplification. In **Supplementary Figure 4**, we present the fidelities of the mapped squeezing operators with different squeezing parameters. Here the target matrix is set to the squeezing operator truncated in $2^{10} \times 2^{10}$ dimensional Hilbert space since it has been very close to the squeezing operator in the finite dimensional Hilbert space. It is shown from **Supplementary Figure 4**a that the fidelities become very low in the case of small N along with increasing the squeezing parameter r. To avoid the low fidelity problem and accurately demonstrate the SPT property with our 4-spins sample, we experimentally prepare the ground state $|G\rangle_s$ of the transformed Hamiltonian $\hat{\mathbb{H}}_s = \hat{S}^\dagger(\tilde{r})\hat{\mathbb{H}}\hat{S}(\tilde{r})$. Then the ground state of system can be obtained by $|G\rangle = \hat{S}(\tilde{r})|G\rangle_s$ with $\hat{S}(\tilde{r})$ being the exact squeezing operator. This method has been used to demonstrate the SPT in the main text, and it effectively utilizes the resource of NMR sample with finite spins.

Here we would like to note that the above post-processing used to calculate the order parameter is theoretically strict. From the perspective of experiment, this post-processing also do not influence the credibility of our results. In **Supplementary Figure 3**a, we present the experimental results with and without post-processing, together with the analytical results in the limit parameter regime, where SPT happens exactly. The main results of our work are to check the recovering of SPT in the case of including A^2 term at the critical point. It is decided by the sudden change of the order parameter at the critical point, i.e., the order parameter suddenly boosting from zero at the critical point. Then, regarding this key point of our work, there is not qualitative difference between the experimental results with and without post-processing, as shown in **Supplementary Figure 3**a. Around the critical point, both of them are approximately consistent with the theoretical prediction in the classical oscillator limit $\Omega/\omega \to \infty$ and $N \to \infty$, where the SPT occurs exactly. The values of order parameter in the superradiant phase are indeed different between the above two cases, since too small Hilbert space is used in our experiment for the squeezing operation with large squeezing parameter (see **Supplementary Figure 4**).

Supplementary Note 5. Initial state preparation

At the beginning, the NMR system is at thermal equilibrium state

$$\hat{\rho}_{\mathrm{eq}} = \frac{\mathbb{1}^{\otimes 4}}{2^4} + \frac{\hbar B_0}{2^4 k_B T}\sum_{i=1}^{4}\gamma_i\hat{\sigma}_z^{(i)} \tag{22}$$

where $\mathbb{1}^{\otimes 4}$ represents the $2^4 \times 2^4$ identity operator, $B_0 = 9.6$ Tesla is the static magnetic field, T is the room temperature 300K, and γ_i is the Gyromagnetic ratio of i-th spin. Note that this thermal equilibrium state is a highly mixed state due to $(\hbar B_0\gamma_i)/(2^4 k_B T) \approx 10^{-5}$. At the beginning of our experiment, we need to initialize the 4-spins system as the pseudo-pure state (PPS),

$$\hat{\rho}_{\mathrm{pps}} = \frac{1-\varepsilon}{16}\mathbb{1}^{\otimes 4} + \varepsilon\, |0000\rangle\,\langle 0000| \tag{23}$$

where $\varepsilon \approx 10^{-5}$ is the polarization. Follow the standard convention in ensemble quantum computing[6], we neglect the identity part in the PPS since it has no influence on NMR signals. From now on, we regard PPS as a true vacuum state.

To prepare the PPS, we use selective-transition approach[7], where some unitary matrices and two field gradient pulses along z direction (Gz) are implemented. Field gradient pulses are used to implement non-unitary operation. Notice that the PPS has a diagonal density matrix, where the first diagonal element has a relatively large value and the others are the same. So, we should redistribute the diagonal elements from equilibrium state while keep the off-diagonal elements as zeros. The first step is applying 14 single-transition unitary operators $\hat{U} = \exp\left(-i\beta_{jk}\hat{u}_{jk}\right)$ to equilibrium state to redistribute the populations, where j and k represent different energy levels and $\hat{u}_{jk} = (|j\rangle\langle k| + |k\rangle\langle j|)/2$. The transition angle β_{jk} is optimized by numerical search procedure. After these operations, the diagonal elements are well distributed, but many undesired off-diagonal elements (coherence terms) come out. Then a following Gz pulse is used to eliminate the coherence terms except the zero-quantum coherences of three homonuclear ^{19}F spins. Next, these zero coherence terms are transferred to other no-zero coherence terms,

followed by the second Gz pulse. Now the PPS is prepared and the full tomography[8] results show the fidelity between the experimental PPS and the pure state $|0000\rangle\langle 0000|$ is over 0.99. Here the fidelity between states $\hat{\rho}_1$ and $\hat{\rho}_2$ is defined as

$$F(\hat{\rho}_1, \hat{\rho}_2) = \frac{\mathrm{tr}(\hat{\rho}_1\hat{\rho}_2)}{\sqrt{\mathrm{tr}\left(\hat{\rho}_1^2\right)\mathrm{tr}\left(\hat{\rho}_2^2\right)}}. \tag{24}$$

Supplementary Note 6. Measurements of the ZPF and order parameter

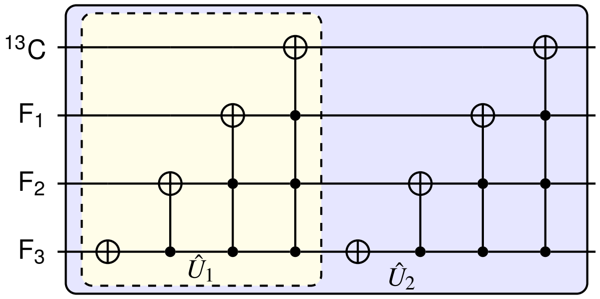

Supplementary Figure 5. **Measurement circuits.** The quantum circuit for operations $\hat{U}_1$ (dashed box) and $\hat{U}_2$ ($\hat{U}_2 = \hat{U}_1^2$).

The ZPF of simulated field and the order parameters of SPT are measured in our experiment. ZPF is defined as ZPF $= \sqrt{\langle\hat{x}^2\rangle - \langle\hat{x}\rangle^2}$ with $\hat{x} = \left(\hat{a} + \hat{a}^\dagger\right)/2$. In the ZPF experiments, we use four spins including ^{13}C nucleus and three ^{19}F nuclei to simulate the truncated boson space of 16×16 dimensions. We first prepare the squeezing vacuum states by applying the squeezing operator to the PPS and then measure $\mathrm{tr}(\hat{\rho}\hat{x}^2)$, $\mathrm{tr}(\hat{\rho}\hat{x})$ respectively. According to our mapping proposal, $\hat{x}$ is described in the mapped basis as

$$\hat{a} + \hat{a}^\dagger = \begin{array}{c} \\ |0\rangle \\ |1\rangle \\ |2\rangle \\ |3\rangle \\ \vdots \\ |15\rangle \end{array} \begin{array}{c} \begin{array}{cccccc} \langle 0| & \langle 1| & \langle 2| & \langle 3| & \cdots & \langle 15| \end{array} \\ \begin{pmatrix} 0 & 1 & 0 & 0 & \cdots & 0 \\ 1 & 0 & \sqrt{2} & 0 & \cdots & 0 \\ 0 & \sqrt{2} & 0 & \sqrt{3} & \cdots & 0 \\ 0 & 0 & \sqrt{3} & 0 & \cdots & 0 \\ \vdots & \vdots & \vdots & \vdots & \cdots & \sqrt{15} \\ 0 & 0 & 0 & \cdots & \sqrt{15} & 0 \end{pmatrix} \end{array} \tag{25}$$

which means

$$\begin{aligned} \hat{a} + \hat{a}^\dagger &= \sum_{n=0}^{15} \sqrt{n+1}|n+1\rangle\langle n| + h.c. \\ &= \sqrt{1}|1\rangle\langle 0| + \sqrt{2}|2\rangle\langle 1| + \sqrt{3}|3\rangle\langle 2| + \cdots + \sqrt{15}|15\rangle\langle 14| + h.c. \\ &= \sqrt{1}|0001\rangle\langle 0000| + \sqrt{2}|0010\rangle\langle 0001| + \sqrt{3}|0011\rangle\langle 0010| + \cdots + \sqrt{15}|1111\rangle\langle 1110| + h.c. \end{aligned} \tag{26}$$

To obtain $\mathrm{tr}(\hat{\rho}\hat{x}) = \frac{1}{2}\left\langle \hat{a} + \hat{a}^\dagger \right\rangle$, we use two readout operators: identity operator and $\hat{U}_1 = \sum_{n=0}^{14} |n\rangle\langle n+1| + |15\rangle\langle 0|$ operator. Specifically, we first read out directly from the NMR spectra of the fourth spin to obtain the expectations of the first, third, fifth . . . terms in Eq. (26) (the single coherence terms). Next we use the $\hat{U}_1$ readout operator to transfer the left terms in Eq. (26) to the corresponding single coherence terms, e.g., $\hat{U}_1|0010\rangle\langle 0001|\hat{U}_1^\dagger = |0001\rangle\langle 0000|$, whose expectations are then obtained by reading the NMR spectra of the fourth spin. An effective quantum circuit to implement $\hat{U}_1$ is shown in **Supplementary Figure 5**, and a linear combination of the above obtained expectations gives the value of $\mathrm{tr}(\hat{\rho}\hat{x})$.

The scheme to obtain $\mathrm{tr}\big(\hat{\rho}\hat{x}^2\big)$ is similar. Note that $\hat{x}^2 = (\hat{a}^{\dagger 2} + \hat{a}^2 + \hat{a}^\dagger\hat{a} + \hat{a}\hat{a}^\dagger)/4$, and we have

$$\hat{a}^2 + \hat{a}^{\dagger 2} = \begin{array}{c} \\ |0\rangle \\ |1\rangle \\ |2\rangle \\ |3\rangle \\ \vdots \\ |15\rangle \end{array}\begin{array}{c} \begin{array}{cccccc} \langle 0| & \langle 1| & \langle 2| & \langle 3| & \cdots & \langle 15| \end{array} \\ \begin{pmatrix} 0 & 0 & \sqrt{1\times 2} & 0 & \cdots & 0 \\ 0 & 0 & 0 & \sqrt{2\times 3} & \cdots & 0 \\ \sqrt{1\times 2} & 0 & 0 & 0 & \cdots & 0 \\ 0 & \sqrt{2\times 3} & 0 & 0 & \cdots & \sqrt{14\times 15} \\ \vdots & \vdots & \vdots & \vdots & \cdots & 0 \\ 0 & 0 & \cdots & \sqrt{14\times 15} & 0 & 0 \end{pmatrix} \end{array} \tag{27}$$

which means

$$\begin{aligned} \hat{a}^2 + \hat{a}^{\dagger 2} &= \sum_{n=0}^{13} \sqrt{(n+1)\times(n+2)}|n+2\rangle\langle n| + h.c. \\ &= \sqrt{1\times 2}|2\rangle\langle 0| + \sqrt{2\times 3}|3\rangle\langle 1| + \sqrt{3\times 4}|4\rangle\langle 2| + \cdots + \sqrt{14\times 15}|15\rangle\langle 13| + h.c. \\ &= \sqrt{1\times 2}|0010\rangle\langle 0000| + \sqrt{2\times 3}|0011\rangle\langle 0001| + \sqrt{3\times 4}|0100\rangle\langle 0010| + \cdots + \sqrt{14\times 15}|1111\rangle\langle 1101| + h.c. \end{aligned} \tag{28}$$

Similar to the case of measuring $\langle \hat{a} + \hat{a}^\dagger\rangle$, we use two readout operators, i.e., identity operator and $\hat{U}_2 = \sum_{n=0}^{13} |n\rangle\langle n+2| + |14\rangle\langle 0| + |15\rangle\langle 1|$ operator, to obtain the $\big\langle \hat{a}^2 + \hat{a}^{\dagger 2}\big\rangle$. Specifically, we first read out directly from the NMR spectra of the third spin to obtain the expectations of the single coherence terms of the third spin in Eq. (28). Next we use the $\hat{U}_2$ readout operator to transfer the left terms in Eq. (28) to the corresponding single coherence terms of the third spin, e.g., $\hat{U}_2|0100\rangle\langle 0010|\hat{U}_2^\dagger = |0010\rangle\langle 0000|$, whose expectations are then obtained by reading the NMR spectra of the third spin. Combining the observed expectations with the coefficients in Eq. (28), the value of $\big\langle \hat{a}^2 + \hat{a}^{\dagger 2}\big\rangle$ is obtained. Moreover, another four readout pulses $\hat{R}_y^{(i)}(\pi/2) = \exp\Big(-i\pi/4\hat{\sigma}_y^{(i)}\Big)$ are needed to reconstruct the diagonal elements to obtain $\big\langle \hat{a}^\dagger\hat{a} + \hat{a}\hat{a}^\dagger\big\rangle$. Combining all the measured expectations gives $\mathrm{tr}\big(\hat{\rho}\hat{x}^2\big)$.

In the ZPF experiment, we also perform full state tomography to reconstruct the density matrices of the squeezed vacuum states. In NMR setup, we first obtained only the deviation of the density matrix $\rho_\Delta = \rho - \mathbb{1}/2^n$ in NMR tomography, which can't be regarded as a quantum state. Therefore, we used the post-processing procedure to reach the density matrices in Fig. 4 by introducing the constraints of the normalization $\mathrm{Tr}(\rho) = 1$, the hermiticity $\rho = \rho^\dagger$ and the positive semi-definiteness $\rho \geq 0$. All of these constrains were realized by CVX toolbox in Matlab. Therefore, the reconstructed matrix will satisfy all the requirements of the density matrix of a quantum state. **Supplementary Figure 6** shows the obtained density matrices, and the corresponding Wigner functions are drawn in Fig. 2 in the main text, which clearly illustrate the antisqueezing effect.

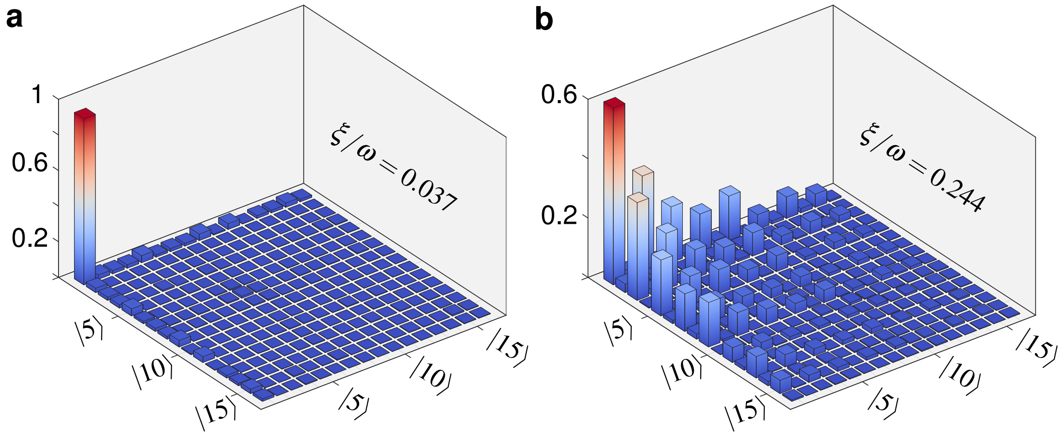

Supplementary Figure 6. The tomography data of the squeezing vacuum states corresponding to two Wigner functions in Fig. 2. a, $\xi/\omega = 0.037$. **b,** $\xi/\omega = 0.244$.

Next, let us describe the experimental details of measuring the order parameter of SPT. In the SPT experiments, we use the ^{13}C nucleus to simulate the two-level system and three ^{19}F nuclei to simulate the truncated boson space of 8×8 dimensions. As mentioned in Methods, the order parameters of bosonic field is:

$$\Phi = (\omega/\Omega)\cosh(2\tilde{r})\langle \hat{a}^\dagger\hat{a}\rangle_s - (1/2)\sinh(2\tilde{r})\Big(\langle \hat{a}^{\dagger 2}\rangle_s + \langle \hat{a}^2\rangle_s\Big) + \sinh^2\tilde{r}, \tag{29}$$

which can be obtained by measuring $\langle \hat{a}^\dagger \hat{a} \rangle_s$ and $\langle \hat{a}^{\dagger 2} \rangle_s + \langle \hat{a}^2 \rangle_s$ with ground state $|G\rangle_s$. To get $\langle \hat{a}^\dagger \hat{a} \rangle_s$, we separately apply a $\pi/2$ readout pulse $\hat{R}_y(\pi/2) = \exp(-i\pi/4\hat{\sigma}_y)$ to three ^{19}F spins after a pulsed field gradient and then measuring the resulting NMR spectrum of the corresponding ^{19}F spin. The NMR spectra are shown in the lines 1-3 of **Supplementary Figure 7**b. For measuring $\langle \hat{a}^{\dagger 2} \rangle_s + \langle \hat{a}^2 \rangle_s$, we need two readout spectra. Notice that

$$\begin{aligned} \hat{a}^{\dagger 2} + \hat{a}^2 = & \sum_{l,m\in\{0,1\}} \sqrt{c_{lm}(c_{lm}-1)}\, |l\rangle\langle l| \otimes \hat{\sigma}_x^{(2)} \otimes |m\rangle\langle m| \\ & + \sqrt{3\times 4}\,(|100\rangle\langle 010| + |010\rangle\langle 100|) + \sqrt{4\times 5}\,(|101\rangle\langle 011| + |011\rangle\langle 101|)\,. \end{aligned} \tag{30}$$

The expectation of the first term is obtained by reading out the second ^{19}F spin directly, and the corresponding spectrum is shown in the line 5 of **Supplementary Figure 7**b. For the last two terms, i.e., $|100\rangle\langle 010| + |010\rangle\langle 100|$ and $|101\rangle\langle 011| + |011\rangle\langle 101|$, we can firstly transfer them into the observable of the second ^{19}F spin (namely $\hat{\sigma}_x^{(2)}$) by applying the designed operation $\hat{U}_2' = \sum_{n=0}^{5} |n\rangle\langle n+2| + |6\rangle\langle 0| + |7\rangle\langle 1|$ to the bosonic field. Subsequently, the corresponding expectations are obtained by measuring the second ^{19}F spin again. The corresponding spectrum is the line 4 of **Supplementary Figure 7**b. Here the operation $\hat{U}_2'$ can be implemented by the quantum circuit shown in **Supplementary Figure 5** or an equivalent GRAPE pulse.

ξ	$\mathbb{D}_{\text{tot}}$	$\mathbb{D}_{\text{pps}}$	$\mathbb{D}_{\text{deco}}$	Error bar
0.001	0.063	0.044	0.084	0.030
0.037	0.090	0.037	0.101	0.029
0.072	0.173	0.026	0.102	0.028
0.108	0.139	0.019	0.085	0.024
0.143	0.046	0.028	0.092	0.023
0.179	0.064	0.040	0.080	0.020
0.214	0.121	0.049	0.061	0.016
0.226	0.054	0.059	0.054	0.016
0.238	0.108	0.049	0.004	0.013
0.244	0.088	0.024	0.037	0.010
0.249	0.066	-0.030	0.008	0.007

$\tilde{\lambda}$	$\mathbb{D}_{\text{tot}}$	$\mathbb{D}_{\text{pps}}$	$\mathbb{D}_{\text{deco}}$	Error bar
0.2	-0.0334	-0.006	-0.048	0.006
0.25	-0.0378	-0.002	-0.020	0.002
0.3	-0.0085	-0.004	-0.018	0.001
0.35	-0.0066	-0.002	-0.020	0.001
0.4	0.0004	-0.008	-0.016	0.001
0.45	-0.0018	-0.003	-0.008	0.001
0.5	0.0097	0.000	-0.004	0.001
0.63	0.0083	0.000	0.001	0.001
0.8	0.0130	-0.001	0.002	0.001
1	0.0097	0.001	0.003	0.001
1.2	0.0089	0.001	0.004	0.001

Supplementary Table 1. The left table is the errors of ZPF (Fig. 2 of the main text). The right table is the errors of the order parameters (Fig. 3 of the main text), where $\Omega/\omega = 50$. The $\mathbb{D}_{\text{tot}}$ column is the total error of ZPF (or the order parameter), which is defined as the deviation of experimental results from theory results. The 3rd and 4th columns corresponding to the errors influenced by the imperfect experimental PPS and decoherence, respectively. The last column represents the standard deviations of ZPF (or the order parameter) coming from the fitting errors.

Supplementary Note 7. Experimental errors and antisqueezing enhanced ZPF

The errors in our experiment include the deviation of experimental data from theoretical results and the variances of experimental data (i.e., errors bar). Different experimental errors on measuring the ZPF and order parameter are shown in the left and right parts of **Supplementary Table 1**, respectively. Without loss the generality, we choose the case of measuring ZPF as example (i.e., the errors in Fig. 2 of the main text) to discuss different experimental errors. On the one hand, the deviation mainly comes from the decoherence and the initial state imperfection. Firstly, we numerically apply ideal squeezing operator to the experimentally reconstructed initial state $\hat{\rho}_{\text{pps}}$, followed by an ideal readout. Then we get the ZPF value which is mainly influenced by the initial state imperfection. The deviations between the influenced ZPF and theoretical ZPF are shown in the third column of **Supplementary Table 1**. Secondly, the effects of decoherence in NMR system can be described by the generalized amplitude damping channel ϵ_{GAD} and the phase damping channel ϵ_{PD}[9]. For small duration Δt, the errors of the phase damping channel is involved by $\hat{\rho} \to \epsilon_{PD}^{(4)} \circ \epsilon_{PD}^{(3)} \circ \epsilon_{PD}^{(2)} \circ \epsilon_{PD}^{(1)}(\hat{\rho})$, where $\epsilon_{PD}^{(i)}(\hat{\rho}) = (1-p_i)\hat{\rho} + p_i \hat{\sigma}_z^{(i)} \hat{\rho} \hat{\sigma}_z^{(i)}$, and $p_i = \frac{1}{2}\left[1 - \exp\left(-\Delta t/T_2^{(i)}\right)\right]$ $(i = 1,2,3,4)$. Here $T_2^{(i)}$ is the decoherence parameter which can be found in Fig. 1 of the main text. Similarly, the generalized amplitude damping error is characterized as $\hat{\rho} \to \epsilon_{GAD}^{(4)} \circ \epsilon_{GAD}^{(3)} \circ \epsilon_{GAD}^{(2)} \circ \epsilon_{GAD}^{(1)}(\hat{\rho})$ and it is

calculated by $\epsilon_{GAD}^{(i)}(\hat{\rho}) = \sum_s E_s^{(i)} \hat{\rho} E_s^{(i)\dagger}$, where

$$E_1^{(i)} = \sqrt{p}\begin{pmatrix} 1 & 0 \\ 0 & \sqrt{1-\eta^{(i)}} \end{pmatrix}, E_2^{(i)} = \sqrt{1-p}\begin{pmatrix} 0 & 0 \\ \sqrt{\eta^{(i)}} & 0 \end{pmatrix},$$
$$E_3^{(i)} = \sqrt{1-p}\begin{pmatrix} \sqrt{1-\eta^{(i)}} & 0 \\ 0 & 1 \end{pmatrix}, E_4^{(i)} = \sqrt{p}\begin{pmatrix} 0 & \sqrt{\eta^{(i)}} \\ 0 & 0 \end{pmatrix}, \tag{31}$$

with $\eta^{(i)} = 1 - \exp\left(-\Delta t/T_1^{(i)}\right)$, $p \approx 1/2$, and $T_1^{(i)}$ is the decoherence parameter shown in Fig. 1 of the main text.

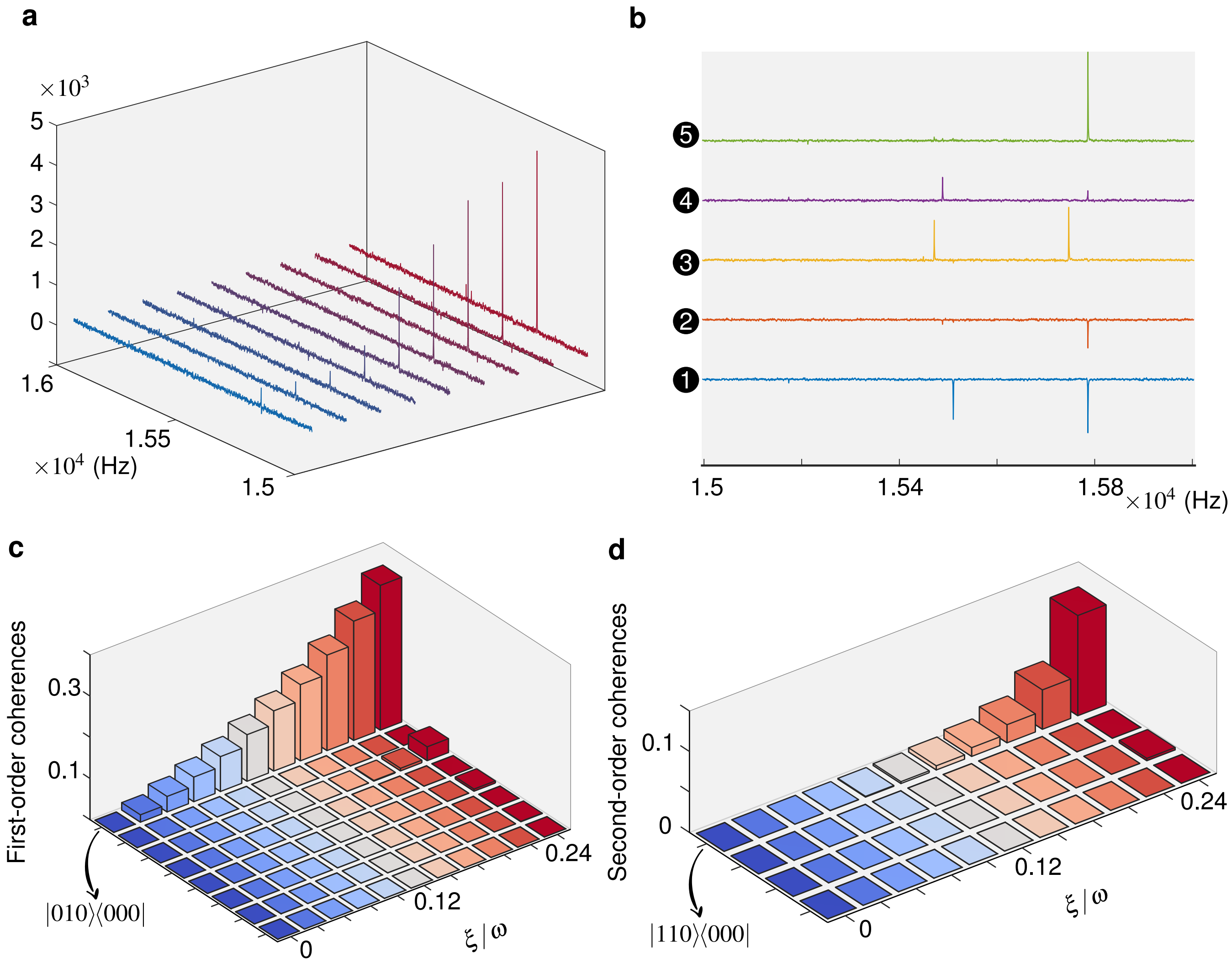

Supplementary Figure 7. The NMR spectra and coherence enhancement by squeezing effect. a, The readout spectra for obtaining the ZPF of system. As the antisqueezing effect increasing, the spectral amplitude grows. **b,** Five NMR readout spectra for measuring order parameters of SPT. Spectra (1-3) are obtained by applying $\pi/2$ pulse along y axis to three ^{19}F spins. Spectrum 4 corresponds to read out the fourth qubit after applying operation $\hat{U}_1$ to the system. Reading out directly the fourth spin gives Spectrum 5. **c,** The third qubit's first-order coherences (like $|{*}1{*}\rangle\langle{*}0{*}|$) in different squeezing parameters; **d,** The second and third qubits' second-order coherences (like $|11{*}\rangle\langle 00{*}|$) in different squeezing parameters.

With ideal initial state and measurement, the numerical simulation shows that the decoherence has much influence on the final ZPF, as shown in the fourth column of Supplementary Table 1. On the other hand, the readout errors shown in the last column of Supplementary Table 1 mainly come from the statistical fluctuation of the NMR spectra. In our experiment, the peak intensities are obtained by fitting the NMR spectra to a sum of Lorentz functions. The results of fitting indicate that these intensities have

about 0.002 standard error, which will contribute to a standard error of the ZPF through error propagation formula. The variance errors of ZPF are

$$\Delta^2_{\mathrm{ZPF}} = \frac{1}{4}\frac{1}{\mathrm{ZPF}^2}\Delta^2(\mathrm{tr}\left(\hat{\rho}\hat{x}^2\right)) + \frac{1}{\mathrm{ZPF}^2}\,\mathrm{tr}(\hat{\rho}\hat{x})\Delta^2(\mathrm{tr}(\hat{\rho}\hat{x})), \tag{32}$$

where $\Delta^2[\mathrm{tr}(\hat{\rho}\hat{x}^2)]$ and $\Delta^2[\mathrm{tr}(\hat{\rho}\hat{x})]$ are the variance errors of $\mathrm{tr}(\hat{\rho}\hat{x}^2)$ and $\mathrm{tr}(\hat{\rho}\hat{x})$, respectively, which are obtained from the errors of peak intensities through linear error propagation. Interesting conclusion is obtained from Eq. (32) and the last column of **Supplementary Table 1**, i.e., the variance (or errors bar) of ZPF decreases as increasing the antisqueezing effect. This originally comes antisuqeezing enhanced signal-to-noise ratio (SNR) of the NMR spectra. As shown in **Supplementary Figure 7** a, a typical NMR readout spectra shows that the signal peak increase as increasing the antisqueezing effect, whereas the noises almost keep a constant. This conclusion is consistent with Fig. 2 of the main text.

Let's further discuss the mechanism of ZPF enhancement in Fig. 2. The NMR spectra that contribute most to ZPT are displayed in **Supplementary Figure 7**a, which are closely related with the coherence term of the simulated oscillator. The quantum coherence of the squeezed vacuum state will be enhanced as increasing the antisqueezing strength, which can be further analytically proved by the mathematical form of squeezed vacuum state

$$S(r)\,|0\rangle = \frac{1}{\sqrt{\cosh r}}\sum_{n=0}^{\infty}(-\tanh r)^n\,\frac{\sqrt{(2n)!}}{2^n n!}\,|2n\rangle\,. \tag{33}$$

By means of the spin-to-oscillator mapping scheme (see the methods part of main text), we have the mapping $|0\rangle \mapsto |0000\rangle$, $|2\rangle \mapsto |0010\rangle$, $|6\rangle \mapsto |0110\rangle$. Thus we easily get the amplitude of the first-order coherence (like $|000\rangle\langle 010|$) and second-order coherence (like $|000\rangle\langle 110|$) as:

$$A_{1\mathrm{st}} = \frac{-\tanh r}{\sqrt{2}\cosh r}, \quad A_{2\mathrm{nd}} = \frac{-\sqrt{5}\tanh^3 r}{4\cosh r}, \tag{34}$$

where $r = \frac{1}{4}\ln\left(1 - 4\frac{\xi}{\omega}\right)$. Then we can calculate the analytical amplitudes of first- and second-order coherences in different squeezing parameters. With the increasing of the squeezing parameter r, both $A_{1\mathrm{st}}$ and $A_{2\mathrm{nd}}$ grow, which is plotted in **Supplementary Figure 7** c and d. This leads to the enhancement of the zero-point fluctuation $\mathrm{ZPF} = \sqrt{\langle\hat{x}^2\rangle - \langle\hat{x}\rangle^2}$ with $\hat{x} = \left(\hat{a} + \hat{a}^\dagger\right)/2$ and $\hat{x}^2 = (\hat{a}^{\dagger 2} + \hat{a}^2 + \hat{a}^\dagger\hat{a} + \hat{a}\hat{a}^\dagger)/4$.

References

1. Hwang, M. J., Puebla, R. & Plenio, M. B. Quantum phase transition and universal dynamics in the rabi model. *Phys. Rev. Lett.* **115**, 180404 (2015).
2. Ourjoumtsev, A., Jeong, H., Tualle-Brouri, R. & Grangier, P. Generation of optical 'schrodinger cats' from photon number states. *Nature* **448**, 784–6 (2007).
3. Wootters, W. K. A wigner-function formulation of finite-state quantum mechanics. *Ann. Phys.* **176**, 1–21 (1987).
4. Cai, M. L. *et al.* Observation of a quantum phase transition in the quantum rabi model with a single trapped ion. *Nat. Commun.* **12**, 1126 (2021).
5. Nataf, P. & Ciuti, C. No-go theorem for superradiant quantum phase transitions in cavity qed and counter-example in circuit qed. *Nat. Commun.* **1**, 72 (2010).
6. Gershenfeld, N. A. & Chuang, I. L. Bulk spin-resonance quantum computation. *Science* **275**, 350–6 (1997).
7. Peng, X. H. *et al.* Preparation of pseudo-pure states by line-selective pulses in nuclear magnetic resonance. *Chem. Phys. Lett.* **340**, 509–516 (2001).
8. Lee, J. S. The quantum state tomography on an nmr system. *Phys. Lett. A* **305**, 349–353 (2002).
9. Vandersypen, L. M. *et al.* Experimental realization of shor's quantum factoring algorithm using nuclear magnetic resonance. *Nature* **414**, 883–7 (2001).
